# MicroRNAs Associated with IgLON Cell Adhesion Molecule Expression

**DOI:** 10.3390/cimb46070456

**Published:** 2024-07-19

**Authors:** Marco Salluzzo, Clara Vianello, Francesca Flotta, Roberto Rimondini, Lucia Carboni

**Affiliations:** 1 Department of Pharmacy and Biotechnology, Alma Mater Studiorum University of Bologna, 40126 Bologna, Italy; marco.salluzzo@unibo.it; 2 Department for Life Quality Studies, Alma Mater Studiorum University of Bologna, 47921 Rimini, Italy; clara.vianello2@unibo.it; 3 Department of Medical and Surgical Sciences, Alma Mater Studiorum University of Bologna, 40126 Bologna, Italy; francesca.flotta2@unibo.it (F.F.); roberto.rimondini@unibo.it (R.R.)

**Keywords:** miRNA, long non-coding RNA, LSAMP, LSAMP-AS1, OPCML, neurotrimin, NEGR1, IgLON5, cell adhesion molecules

## Abstract

The IgLON family of cell adhesion molecules consists of five members (LSAMP, OPCML, neurotrimin, NEGR1, and IgLON5) discovered as supporters of neuronal development, axon growth and guidance, and synapse formation and maintenance. Tumour suppression properties have recently been emerging based on antiproliferative effects through the modulation of oncogenic pathways. Available evidence endorses a role for non-coding RNAs or microRNAs as relevant controllers of IgLON molecule expression that can impact their critical physiological and pathological roles. Current findings support a function for long non-coding RNAs and microRNAs in the modulation of LSAMP expression in cell senescence, cancer biogenesis, addiction, and pulmonary hypertension. For OPCML, data point to a role for several microRNAs in the control of tumorigenesis. MicroRNAs were detected in neurotrimin-mediated functions in cancer biogenesis and in Schwann cell responses to peripheral nerve injury. For NEGR1, studies have mainly investigated microRNA involvement in neuronal responses to ischaemic injury, although data also exist about tumorigenesis and endothelial cell dysfunction. For IgLON5, information is only available about microRNA involved in myocardial infarction. In conclusion, despite much information being still missing and further research needed, the emerging picture favours a model in which non-coding RNAs exert a crucial role in modulating IgLON expression, ultimately affecting their important physiological functions.

## 1. Introduction

**Figure 1:**
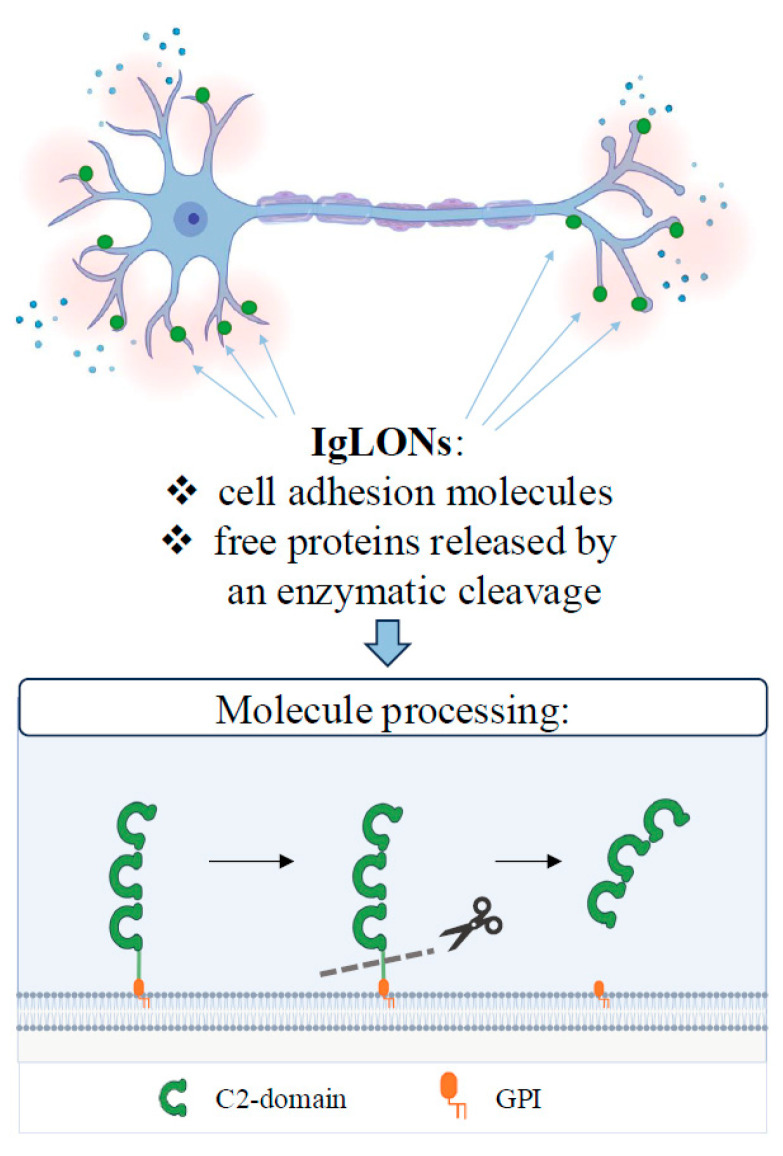
Representative image of IgLON localisation in neurons (green) and schematic illustration resuming the main steps of their enzymatic processing.

The IgLON family belongs to the immunoglobulin (Ig)-like family of cell adhesion molecules. Cell adhesion molecules are located on the cell membrane to mediate interactions with molecules belonging to other cells or to the extracellular matrix, thus contributing to essential biological functions, including adhesion, recognition, differentiation, and migration. Among cell adhesion molecules, the Ig-like family is characterised by the presence of at least one immunoglobulin domain. In particular, the subgroup of IgLON members contains three C2 immunoglobulin domains in the N-terminal portion (Figure 1). Since IgLONs lack a transmembrane portion, anchoring to the cellular membrane is granted by a glycosylphosphatidylinositol (GPI) segment (Figure 1). All IgLONs are glycosylated in different portions of the molecule (Figure 1) and form homomeric or heteromeric bonds in cis or in trans to perform their functions [1]. The family encompasses five members that were differently named by multiple discoverers and subsequently renamed for the sake of clarity (Table 1). The first identified protein was dubbed limbic system-associated membrane protein (LAMP) [2], but subsequently renamed LSAMP to distinguish it from the lysosomal-associated membrane protein. It was specifically expressed in neurons of the limbic system during development with a function in axonal growth and pathway formation [2,3,4]. Opioid-binding protein/cell adhesion molecule-like (OPCML, also known as OBCAM) was identified due to its ability to bind opioid receptors, in addition to the cell adhesion molecule functions [5,6]. The third cloned member, neurotrimin, (NTM) shares structural features with the other proteins [7] as well as the ability to dimerise and being shed to a truncated but functional form by the action of proteases [8]. Neuronal growth regulator 1 (NEGR1) was previously termed KILON/neurotractin [9,10], and shares the family properties as a promoter of nervous system development and maintenance [11]. The most recent addition, IgLON5, is associated with autoimmune encephalitis [12].

The IgLON family is recognised as a critical player in the development of the central nervous system, neurite formation and extension, synapse creation and maintenance, and neuroprotection [13]. Not surprisingly, evidence is accumulating of critical involvement in neuropsychiatric and neurodegenerative disorders [14]. In addition, recent findings support a prominent function as tumour suppressors [15,16]. Since much is still unknown about the mechanisms regulating their expression, this review is aimed at collecting available information about the regulatory mechanisms involving non-coding RNAs.

## 2. MicroRNA Overview: Biogenesis, Regulatory Properties, and Nomenclature Tips

**Figure 2:**
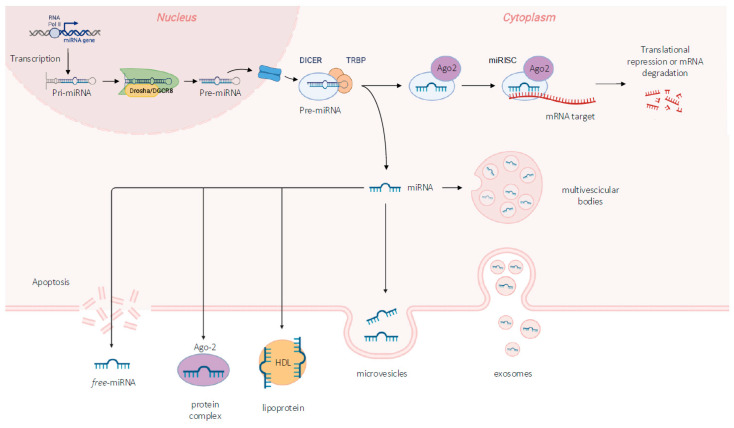
Scheme of microRNA biogenesis pathway and secretion.

A microRNA, or miRNA, is a single-stranded RNA consisting of 20–22 nucleotides that acts as a regulator of gene expression in the post-transcriptional phase [17]. The first miRNA was discovered in 1993 by Victor Ambros, Rosalind Lee, and Rhonda Feinbaum while they were studying lin-4, a gene known to control the timing of *C. elegans* larval development [18]. Since their discovery in 1993 [18], there was a rapid and exponential accumulation of evidence that reported the fundamental role of these non-coding molecules in several biological processes acting at post-transcriptional level. Regularly, miRNAs bind to a complementary sequence in the 3′-untranslated region (UTR) of mRNA, causing mRNA silencing or degradation [19,20]. Their biogenesis pathway takes place in the cell nucleus, where polymerase II transcribes most miRNAs starting from DNA sequences into primary transcripts (pri-miRNAs). Pri-miRNAs are then processed into shorter precursor miRNAs (~65–70 nt) with a characteristic stem–loop secondary structure, a key feature of the following step requiring the microprocessor complex. This complex consists in two proteins: DiGeorge syndrome critical region 8 (DGCR8), which recognises and binds the stem–loop structures, and Drosha, which is the ribonuclease (RNase) III enzyme responsible for cutting off the pre-miRNA hairpins from primary transcripts [21,22]. Subsequently, the Exportin 5 (XPO5)–Ran-GTP complex carries the pre-miRNA molecule via the nuclear pore from the nucleus to the cytoplasm, where it is immediately processed by Dicer into a 22-nucleotide-long RNA duplex with 2-nucleotide 3′-overhanging ends. Dicer is a cytoplasmatic enzyme belonging to the RNase III family endowed with a crucial role in the mature miRNA biogenesis system in producing the mature form of miRNA [21,23]. Dicer activity is supported by the RNA-binding protein (TRBP), which coordinates pre-miRNA recognition. TRBP recruits double-stranded RNAs (dsRNAs) without selectivity, but with a transitory binding. Whereas non-canonical substrates are quickly released by TRBP, canonical pre-miRNAs remain firmly bound and transferred to Dicer for cleavage [24]. This selective loading by Dicer–TRBP promotes efficient RNA processing despite the RNA-crowded environment. Afterwards, the RNA duplexes are loaded into Argonaute proteins with the support of the HSC70–HSP90 chaperone machinery, which promotes the conversion of the Argonaute protein conformation from a closed to a more open structure receptive to binding RNA duplexes [24]. The Argonaute complex becomes a functional miRNA-induced silencing complex (miRISC) only after the release of one of the two single miRNA strands (sense strands). On the other hand, the single-stranded miRNA encompassed inside the miRISC complex (antisense strand, named guide RNA) can recognise and bind mRNA targets, causing their degradation or inhibition [24]. The selection of the guide RNA is related to the strand stability and clarifies the reason that the antisense strand remains tied to the miRISC complex. Indeed, RISC complex proteins support the leading strand by ensuring its stability. Another member of the complex is Ago2, a protein with an intrinsic endonuclease activity that specifically degrades target RNAs [24]. In most cases, miRNAs interact with the 3′-UTR of target mRNAs to induce their degradation and translational repression [23]. The strategy of gene expression regulation is based on the degree of complementarity between the mRNA 3′-UTR and miRNA seed sequence. The seed sequence represents a conserved region with a higher number of pairing nucleotides, from position 2 to 5 at the 5′-end of the mature miRNA, providing process specificity. When the base pairing is perfect, the complex mediates the degradation of the target mRNA; otherwise, downregulation of translation is induced [19]. The interaction of miRNAs with their target genes is dynamic and dependent on many factors, such as miRNA subcellular location, the abundance of miRNAs and target mRNAs, and the affinity of miRNA–mRNA interactions. miRNAs can be actively secreted into extracellular fluids and transported to target cells via vesicles, such as exosomes, or by binding to proteins, including Argonautes (Figure 2). The most common forms through which extracellular RNAs including miRNA are present in fluids are essentially four: freely circulating, bound to specific proteins, associated with lipoproteins, or enclosed in extracellular vesicles (Figure 2) [25].

The lipid membrane forming exosomes and microvesicles creates a safe microenvironment that protects miRNAs. Hence, this strategy allows miRNAs to circulate into body fluids without being damaged and thus constitute a communication system between cells. On the other hand, passive extrusion mechanisms known as cell fragmentation processes lead to the release of free circulating miRNA [25]. Due to their localisation and availability, circulating miRNAs are promising diagnostic and prognostic biomarkers for the non-invasive detection of disease [26]. The expression profile of specific miRNAs reflects the development of a variety of diseases in both their early and advanced stages.

In consequence of their role as potential diagnostic and prognostic biomarkers and the availability of high-throughput miRNA identification techniques, our knowledge of these molecules has grown quickly. This has highlighted the importance of establishing a central database and standardizing terminology associated with miRNAs [27]. A uniform system of basic rules for miRNA annotation was proposed over a decade ago [28]; however, the nomenclature was not adopted by all researchers and a significant number of newly discovered miRNAs have not been properly annotated or registered in miRbase. Some publications named miRNAs in a manner that does not follow the original guidelines established by Ambros and his colleagues [28], so we summarise in this review the most important of them. Currently, mature miRNAs are given the ‘miR’ prefix followed by a numerical identifier (e.g., miR-22) often indicating order of naming. The early miRNAs discovered in the research history, such as lin-4 and let-7, are exceptions because they do not contain the ‘miR’ prefix [27]. A different annotation is used to easily distinguish animal miRNAs from plant ones. Animal miRNAs are named with lowercase, italic characters and with a hyphen immediately after the ‘miR’ prefix (e.g., miR-14). Conversely, the names of plant miRNAs are distinguished by the capitalisation of the ‘mir’ prefix and the omission of the hyphen, such as MIR172 [27]. Also, the prefix font is a guide to identify the species of origin. In literature, the annotations MIR, Mir, and mir, are associated with the species of human, mouse, and zebrafish, respectively. Always with the aim of identifying the species name, a three-letter prefix is used. This designation discriminates identical miRNAs named with the same identifier number that derive from different organisms [29]. Letters in miRNA nomenclature are also used for identifying miRNAs that have almost identical sequences, but differ by only one or two nucleotides. Hence, a lowercase letter follows the hyphen immediately after the ‘miR’ annotation, as reported for miR-20a, which would be closely related to miR-20b. A second numerical index, preceded by a hyphen, is used to specify those pre-miRNAs that give rise to mature miRNAs with 100% sequence identity, but that are located in different regions of the genome. For example, the pre-miRNAs pre-miR-7-1 and pre-miR-7-2 lead to an identical mature miRNA (miR-7), but are placed in different genomic regions. Where the mature miRNA originates from opposite ends of the same pre-miRNA, the arm of origin is indicated. The miRNA derived from the 5′ end is thus denoted 5p, while the 3p designation is assigned for those derived from 3′ ends. Currently, the leading scientists in the field have proposed two different miRNA annotation guidelines [28,30]. Overall, informatic tools such as the miRBase website (https://www.mirbase.org/help/#, accessed on 17 June 2024) are available online as reference and for better clarification. It is of great importance that researchers use these guidelines and tools to avoid errors and improve the accuracy of future research.

In the following sections, a list of studies reporting miRNAs associated with each IgLON protein will be reviewed, highlighting the most important discoveries. When available, information is also included regarding another class of non-coding RNAs termed long non-coding RNAs (lncRNAs). lncRNAs are defined as non-coding transcripts of more than 200 nucleotides that are recognised to exert regulatory functions on gene expression [31]. All miRNAs described are summarised in Table 2, indicating the associated IgLON and the effect exerted on their expression.

## 3. LSAMP

In one of the first studies on LSAMP modulations by non-coding RNAs, Jeng et al. [32] investigated the modulation of muscle-specific miRNAs by denervation and reinnervation in a rat model. miR-206 was found to be specifically upregulated by the reinnervation procedure, with the increase lasting for at least 4 months. The combination of results of mRNA expression profiles by whole rat genome oligo microarrays of reinnervated muscle samples combined with in silico predictions indicated LSAMP as a potential target for miR-206, among other genes. These findings suggested a potential regulatory role for miR-206; however, biological samples were not investigated to confirm in silico predictions [32].

A class of lncRNAs is termed natural antisense transcripts because they are complementary to protein-coding transcripts and are supposed to be implicated in gene expression regulation [33]. An investigation aimed at discovering cellular senescence-associated lncRNAs in foetal lung-derived human fibroblasts discovered that LSAMP-AS1, antisense to the LSAMP mRNA, exhibited lower abundance in senescent cells. These findings were confirmed when senescence was induced in the same model with different methods, namely, repeated passages or ionizing radiation exposure. It is conceivable that the altered expression of LSAMP-AS1 may contribute to the senescent phenotype by modulating some among its characteristic traits, including terminal growth arrest, autophagy, cell survival, and DNA damage and repair [34].

The relevance of LSAMP-AS1 in the regulation of cellular processes was confirmed by a study that investigated the differential expression of lncRNAs in laryngeal squamous cell carcinoma or adjacent normal mucosa samples from the Cancer Genome Atlas database. A panel made up of seven upregulated lncRNA, including LSAMP-AS1, was identified, the levels of which were associated with better prognosis based on survival data. A ceRNA network was built that showed direct or indirect regulatory relationships among the seven lncRNAs mediated by 18 miRNAs [35]. In agreement with these data, a similar approach was adopted by Nie et al. [36] in laryngeal cancer samples from the Cancer Genome Atlas, but after specifically focussing on lncRNAs associated with the PI3K–Akt signalling pathway, which is considered central to the regulation of tumour cell apoptosis, nutrient production, angiogenesis, cell growth, and metastasis. This effort allowed the identification of three lncRNAs (LSAMP-AS1, MNX1-AS1, and LINC00330) whose increased levels were suitable as an effective prognostic signature. The panel’s validity was verified in an independent cohort of patients, thus confirming LSAMP-AS1’s critical role in controlling processes like cell proliferation, viability, and migration [36].

In addition to LSAMP-AS1, other lncRNAs were previously named after LSAMP, such as LSAMP-AS2 (now LINC00903: long intergenic non-protein-coding RNA 903), LSAMP-AS3 (TUSC7: tumour suppressor candidate 7), LSAMP-AS4 (LINC00901: long intergenic non-protein-coding RNA 901), and Lnc-LSAMP-1 (LINC03051: long intergenic non-protein-coding RNA 3051, https://www.genenames.org/tools/search/#!/?query=lsamp, accessed on 3 June 2024). In particular, LSAMP-AS3/TUSC7 has been recognised as a potential tumour-suppressing gene that a wealth of data have implicated in many human cancers, including glioma, pancreatic ductal adenocarcinoma, osteosarcoma, oesophageal squamous cell carcinoma, and colorectal, gastric, and breast cancers [37,38]. As already mentioned, a role as a tumour-suppressing gene for LSAMP itself is supported by a large amount of data regarding renal cell carcinoma, osteosarcoma, prostate cancer, glioblastoma, neuroblastoma, and lung cancer [16,39,40,41,42,43,44]. In particular, the characterised relevance in prostate and bladder cancers [40,45,46,47] will require specific attention in future investigations.

Since the role of lncRNAs is only imperfectly understood, a ‘competitive endogenous RNA (ceRNA)’ hypothesis has been proposed to help in interpreting their mechanism of action. This hypothesis posits that both mRNAs and lncRNAs affect gene expression (and final protein translation) by binding to miRNA-binding sites [48]. Thus, lncRNAs can act as endogenous miRNA sponges that competitively bind to a limited pool of miRNAs, thus influencing their availability for binding target genes. Within this frame, a study aimed to discover a ceRNA network within differentially expressed RNAs based on the miRNA-binding sites on both lncRNAs and mRNAs in laryngeal squamous cell carcinoma in the Cancer Genome Atlas database. A relevant role for aberrant LSAMP-AS1 expression was identified within the ceRNA network, suggesting that it represents a critical factor for prognosis, as evaluated in survival analysis data. In particular, upregulated LSAMP-AS1 in tumour samples directly inhibited miR-375, which is considered a tumour suppressor [49]. CeRNA networks were also investigated by Dell’Orco et al. [50] in an effort to identify the role of the RNA-binding protein HuD in substance use disorders. Since HuD is known to regulate mRNAs as well as non-coding RNAs belonging to different groups, differentially expressed RNAs were analysed in the mouse striatum of HuD KO mice, a brain region associated with drug addiction. An interaction analysis was carried out to build ceRNA networks from differentially expressed mRNAs, miRNAs, and circRNAs, a class of non-coding circular RNAs generated by back-splicing [51]. LSAMP appeared to play a critical role in the ceRNA network, as its mRNA was significantly downregulated in HuD KO mice, whereas 30 upregulated miRNAs were predicted to interact with it (miR-1193-3p; miR-129-1-3p; miR-129-2-3p; miR-132-3p; miR-1930-5p; miR-212-3p; miR-297a-3p; miR-297b-3p; miR-297c-3p; miR-3068-3p; miR-3069-3p; miR-3072-3p; miR-3102-3p; miR-326-3p; miR-329-3p; miR-350-3p; miR-369-3p; miR-434-3p; miR-450b-3p; miR-466a-3p; miR-466b-3p; miR-466c-3p; miR-466e-3p; miR-466f-3p; miR-466h-5p; miR-466p-3p; miR-467d-3p; miR-488-3p; miR-700-5p; miR-874-5p) and also predicted to bind circLSAMP. Therefore, LSAMP downregulation was interpreted as a consequence of reduced circLSAMP levels due to HuD absence, which freed the 30 miRNAs, allowing their direct binding to LSAMP mRNA to reduce its expression. Since HuD is implicated in substance use disorders, these findings suggest a critical contribution for LSAMP [50].

A ceRNA network was similarly built in a model of hypoxia-induced pulmonary hypertension established in rats. A differential expression profile of mRNAs, lncRNAs, circRNAs, and miRNAs was used to construct a ceRNA network, where LSAMP downregulation in the disease model sample was associated with miR-541-5p levels, as well as altered levels of lncRNAs (AABR07000398.1-OT1, LINC1589, Hip1-OT1) and a circRNA (circ_0004345). Lower LSAMP levels were confirmed in pulmonary hypertension patients, thus suggesting a potential diagnostic value for this alteration [52].

In addition, LSAMP-AS1 was identified as part of a ceRNA network critical to the ability to overcome hypoxic conditions in pancreatic cancer cells [53]. Data about differential expression of lncRNA, miRNA, and mRNA were obtained from the Cancer Genome Atlas and Gene Expression Omnibus from pancreatic cancer samples divided into two groups based on the expression of hypoxia-related genes. LSAMP-AS1, miR-129-5p, and the mRNA for S100A2 were indicated as the most relevant network members able to regulate hypoxic environments, thus affecting the prognosis [53].

miRNA can be encoded in intergenic regions as well as within protein-coding genes, which are referred to as host genes. miRNA belonging to the latter type are mainly encoded within intronic regions. Berillo et al. [54] studied intronic intergenic miRNA with the aim of identifying their interactions with target proteins involved in tumorigenesis. miR-4447 was identified as a miRNA encoded within *LSAMP*, and binding sites for this miRNA were located in the 3′ or 5′ untranslated regions or in coding regions of 118 genes. In turn, these target genes are host genes for other intronic miRNA that can influence the expression of other target genes, including the original host genes, ultimately leading to the regulation of the initial overexpressed miRNA. Since most host genes encode for proteins participating in cancer development, disruption of this complex self-regulating network is likely to contribute to tumorigenesis [54].

Further clarification about LSAMP’s role in tumour progression came from a study by Zhou et al. [55]. They discovered that tumour-associated neutrophils are able to promote hepatocellular carcinoma formation, growth, and metastasis both in vitro and in vivo through BMP2 and TGF-β release, which induced miR-301b-3p overexpression. In turn, miR-301b-3p was demonstrated to promote tumorigenesis through the suppression of LSAMP and CYLD mRNA expression, which promoted stem cell traits in tumour cells. In addition, survival analyses showed that reduced LSAMP expression was associated with poor prognosis, thus supporting its potential value as prognostic indicator [55].

Ghobadi et al. [56] aimed to identify differentially expressed interacting coding and non-coding RNAs which were able to classify the three major subtypes of adult T-cell leukaemia/lymphoma RNAs. A weighted gene co-expression network analysis was adopted to analyse Gene Expression Omnibus data to identify co-expressed genes specifically characterising disease subtypes. Among them, differentially expressed genes overlapping with targets of differentially expressed miRNA were determined. The study demonstrated that interactions between miR-29b-2-5p and miR-342-3p with LSAMP characterised specifically the ‘acute’ subtype of adult T-cell leukaemia/lymphoma, and that this interaction is likely to be involved in the pathogenetic mechanism of the disease [56]. Similarly, a weighted gene co-expression network analysis was employed by Xu et al. [57] to identify modules of genes associated with the extent of immune cells and stromal cells in the tumour microenvironment of colorectal cancer, because this feature is relevant for prognosis prediction. A risk model was generated that included *LSAMP* as a protective factor, together with another eight genes (*HLX*, *WWTR1*, *PDGFRA*, *RAB3IL1*, *CRIP2*, *ADAM8*, *CCL22*, and *LGALS1*). The mechanism of LSAMP-mediated protection was shown to be based on LSAMP inhibition of the Wnt–β-catenin signalling pathway, which is dysregulated in immune evasion and metastasis of colorectal cancer. In particular, miRNA-200c-3p was identified as a major regulator of *LSAMP* expression by direct interaction with the with *LSAMP* 3′-UTR to inhibit its expression. In addition, HMGA2, a transcription factor belonging to high-mobility group proteins, was shown to induce miRNA-200c-3p increase by binding to its promoter region. Therefore, a crucial role was demonstrated for the HMGA2–miRNA-200c-3p–LSAMP–Wnt pathway in the immune response to colorectal cancer, with significant implications for improved therapeutic responses [57].

## 4. OPCML

OPCML has emerged as a pivotal gene in cancer research, especially for gastric and thyroid cancers, as shown by various bioinformatic analyses and experimental studies. Its dysregulation highlights its potential as a diagnostic biomarker and therapeutic target, paving the way for novel treatment strategies aimed at improving patient outcomes.

A study by Fang et al. [58] discovered that the lncRNA LINC00619 was significantly reduced in gastric cancer samples compared to normal samples, while miR-224-5p was significantly upregulated. The microarray dataset for gastric cancer was obtained from the Gene Expression Omnibus. RNA22 database identified a binding site between LINC00619 and miR-224-5p, which was confirmed by various assays. A binding site between miR-224-5p and OPCML was also predicted using the DIANA database and TargetScan and validated by RT-qPCR, showing that miR-224-5p downregulated OPCML expression. Overexpression of LINC00619 decreased miR-224-5p levels and increased OPCML expression, whereas silencing LINC00619 had the opposite effect. Functional assays revealed that miR-224-5p inhibition reduced cell proliferation and migration and increased apoptosis. These effects were reversed by OPCML silencing or miR-224-5p overexpression. Additionally, genes related to apoptosis (*Bcl-2* and *Bax*) and metastasis (*MMP-2* and *MMP-9*) were similarly affected by miR-224-5p and OPCML [58].

In another study [59], a ceRNA network was constructed by the differential expression profiles of lncRNAs, miRNAs, and mRNAs detected in gastric adenocarcinoma and gastric adenoma samples obtained from the Cancer Genome Atlas database. Among the genes identified, OPCML emerged as significantly downregulated in gastric cancer, consistent with previous experimental validations [58]. The ceRNA network revealed several key interactions, notably the POU6F2-AS2–hsa-mir-137–OPCML axis, where POU6F2-AS2 acted as a competitive sponge for hsa-mir-137, thereby modulating OPCML expression levels [59].

Building on findings about the impact on tumour progression exerted by OPCML’s significant interactions with miRNAs, recent research by He et al. [60] on cholangiocarcinoma identified the lncRNA OPCML intronic transcript 1 (OPCML-iT1) as a potential prognostic biomarker. The dataset of cholangiocarcinoma tissues compared with normal samples was downloaded from the Cancer Genome Atlas database. A ceRNA network was predicted using the miRTarBase database, and OPCML-iT1 was identified as interacting closely with seven miRNAs, including hsa-mir-372, hsa-mir-373, hsa-mir-519d, hsa-mir-184, hsa-mir-205, hsa-mir-506, and hsa-mir-375, mediating the expression of target genes. Moreover, Kaplan–Meier survival analysis indicated that OPCML-iT1 had positive effects on the overall survival time of patients with cholangiocarcinoma [60].

The discovery of *OPCML* as a tumour suppressor gene in epithelial ovarian cancer [61] adds significant perspective to understanding epigenetic alterations in tumours, expanding upon potential diagnostic and therapeutic applications already explored in cholangiocarcinoma. *OPCML* has been identified among several genes subjected to hypermethylation [62,63], suggesting its potential value as a biomarker for subtype-specific diagnosis and prognosis. Moreover, miRNAs like miR-34B and let-7a-3 have been identified as methylation-sensitive, potentially influencing OPCML expression levels.

Finally, a study conducted by Xu et al. [64] utilised RNA expression profiles from the Cancer Genome Atlas and bioinformatic tools to construct a ceRNA network involving mRNAs, lncRNAs, and miRNAs in thyroid cancer. Survival and functional analyses were conducted to elucidate the role of this ceRNA network in thyroid cancer pathogenesis and progression. The ceRNA network predicted OPCML-IT1 (lncRNA) associated with miR-372, miR-373, miR-519d, miR-184, miR-205, and miR-375 as relevant players in thyroid cancer. However, the specific mechanisms involving this interaction remain to be fully elucidated [64]. Despite the recognised role of OPCML epigenetic modulations in prostate and bladder cancers [65,66,67], no information is yet available about the potential impact of miRNA regulation.

## 5. NTM

Recent research has revealed that the regulation of NTM expression by specific miRNAs can profoundly impact various cellular functions, including those related to cancer and neurodegenerative disorders. Several studies examined how miR-758 and miR-182 influence NTM expression, highlighting its broader effects on cellular activities such as cholesterol metabolism, cell proliferation, and migration.

A study by Ramirez et al. [68] showed that miR-758 overexpression led to the downregulation NTM mRNA expression in glioblastoma cells. Since a number of neurodegenerative disorders are linked to disruptions in cholesterol metabolism, the study aimed to explore the potential role of miR-758 in regulating cellular cholesterol efflux in neural cells. Normally, in response to excess cellular cholesterol, transcription factors such as sterol response element-binding proteins and liver X receptors (LXRs) help maintain cholesterol balance [69] and activate genes like ABCA1 [70]. When glioblastoma cells were transfected with miR-758, ABCA1 expression was reduced through post-transcriptional mechanisms, leading to impaired cholesterol efflux. Conversely, miR-758 inhibition increased ABCA1 protein levels, confirming that ABCA1 is regulated by miR-758. Additionally, miR-758 overexpression decreased NTM mRNA expression, indicating that miR-758 may have a broader impact on neuronal processes beyond cholesterol metabolism, given NTM’s role in various neurological functions [68].

Similarly, other miRNAs have been shown to influence NTM in the nervous system. For instance, Yu et al. [71] explored the dynamic expression of miRNAs in regulating Schwann cells after peripheral nerve injury in rats, identifying a notable upregulation of miR-182 that affected the Schwann cell phenotype, proliferation, and migration. miR-182 was specifically selected because of its inhibitory role in cell proliferation and invasion [72]. Further investigation showed that overexpressing miR-182 directly targeted and downregulated NTM expression in Schwann cells at both mRNA and protein levels, resulting in decreased cell proliferation and migration. Conversely, inhibition of miR-182 increased NTM expression and promoted Schwann cell migration, suggesting a post-injury regulatory role of miR-182 [71] NTM is crucial for cell–cell recognition and guidance during nervous system development [73]. Its regulation by miR-182 highlights a mechanism by which miRNAs influence Schwann cell responses to injury, affecting critical processes for nerve regeneration [71].

## 6. NEGR1

Several non-coding RNAs have been validated to interact with NEGR1 mRNA. Most of these associations have been predicted by databases such as TargetScan (https://www.targetscan.org/vert_80/, accessed on 14 June 2024). These bioinformatic tools aided to select the miRNAs predicted to interact with the gene of interest based on the nucleotide bases interacting with the 3′-UTR nucleotide sequence of NEGR1 mRNA. Predicted associations observed between miRNA and NEGR1 have been further investigated, testing their putative interaction in in vitro models. Several studies have underlined the importance of the mutual interaction between NEGR1 mRNA and non-coding RNAs as significant mechanisms regulating neuronal survival and maturation or, conversely, neuronal death.

The first study shedding light on the regulatory RNAs that interact with NEGR1 goes back to 2014 and was aimed at unveiling the different transcriptome profiles of primary neurons at different developmental time points [74]. Primary cortical neuronal cultures were obtained from E15 mouse embryos, and the lncRNA, miRNA, and mRNA expression profiles were analysed at 4, 6, and 8 days in culture. Most expression variations were observed for molecules belonging to neurotrophic signalling, proliferation, and cellular adhesion molecules (CAM and NEGR1 among them). Validation of these findings was further carried out in an in vitro model of ischaemic injury, where primary neurons were exposed to oxygen and glucose deprivation (OGD). Most mRNAs and lncRNAs showed an opposite expression profile compared to the maturation process, confirming their role in neuronal development and survival. Additionally, the expression of miR-377 was further investigated, as it was predicted to bind to multiple NCAM mRNAs, including NCAM1 and NEGR1. The results showed that during neuronal maturation, the levels of miR-377 and NCAM1 and NEGR1 mRNAs were upregulated, while the levels of associated lncRNAs were downregulated. Thus, NEGR1 mRNA expression, as modulated by the both lcnRNAs and miR-377, was proposed as critical for neuronal maturation [74].

In a follow-up of the previous study, the non-coding RNAs regulating NEGR1 gene expression have been investigated in mature mouse primary cortical neuronal cultures [75]. The predicted NEGR1-interacting miRNAs were investigated, and miR-203 showed the highest negative correlation with NEGR1 mRNA expression. Direct interaction between miR-203 and NEGR1 was demonstrated through luciferase assays, which confirmed the inverse regulation between them. Moreover, increased expression of NEGR1 mRNA and protein, as well as increased neurite length, were observed in cells transfected with a miR-203 inhibitor. In contrast, miR-203 overexpression reduced the levels of NEGR1 mRNA and protein and neurite length. An lncRNA, BC048612, which is transcribed from the NEGR1 promoter, was identified as a positive regulator of the expression of NEGR1 itself. Knockdown of BC048612 expression reduced the expression levels of NEGR1 mRNA and protein, which in turn resulted in reduced neurite length in primary neurons. Conversely, opposite effects were obtained when cells were transfected with BC048612. The opposite effects of BC048612 and miR-203 NEGR1 mRNA expression may represent a regulating pathway relevant for neuronal maturation during development. Furthermore, neuronal injury conditions were investigated based on previous evidence [74,76]. In primary neurons treated in OGD conditions, NEGR1 mRNA levels were dramatically downregulated, whereas BC048612 and miR-203 were increased. Moreover, miR-203 overexpression reduced cell viability in culture, suggesting that miR-203 could impact neuronal death through NEGR1 downregulation [75].

Similar results were obtained in another study, where authors studied the mutual interaction between the lncRNA SNHG12, acting as ceRNA, and miR-181a-5p in apoptosis following cerebral ischaemia [77]. In SH-SY5Y cells exposed to OGD conditions, miR-181a-5p levels increased, while SNHG12 decreased. The decrease in cell viability following OGD was correlated with increased miR-181a-5p expression, whilst SNHG12 overexpression decreased apoptosis and augmented cell survival. Of considerable interest, both molecules possess binding sites for NEGR1 mRNA, and miR-181a-5p overexpression reduced the levels of both NEGR1 mRNA and protein. A reduction in NEGR1 levels was observed in OGD-treated cells, resulting in increased apoptosis and necrosis. miR-181a-5p overexpression in OGD cells caused increased cell death, while NEGR1 overexpression inhibited apoptosis. A similar neuroprotective effect was also observed for SNHG12. These results confirmed that miR-181-5a acts as a pro-apoptotic factor and inhibitor of NEGR1 in the regulation of cell death, and further demonstrated a pro-survival capacity for NEGR1 in inhibiting OGD-induced apoptosis [77].

Another study [78] provided additional proof regarding NEGR1’s neuroprotective role in OGD-treated HCN-2 cells in a pathway also involving the circRNA DLG-associated protein 4 (circDLGAP4) and miR-503-3p. After OGD treatment, circDLGAP4 behaved as a sponge for miR-503-3p, and its overexpression increased cell survival and favoured the expression of protective genes, pro-apoptotic genes, and proteins. Moreover, miR-503-3p inhibition promoted cell viability and decreased the levels of inflammatory proteins. Interestingly, both circDLGAP4 and miR-503-3p were shown to be able to bind the NEGR1 promoter, thus contributing to OGD-induced NEGR1 downregulation. miR-503-3p inhibition and NEGR1 overexpression reduced cell death in OGD, while the opposite held for miR-503-3p overexpression and NEGR1 [78].

The interaction between NEGR1 and miR-576-5p was validated by dual-luciferase assays in colon adenocarcinoma cells. miR-576-5p was overexpressed in tumour cells, with a role in promoting the malignant properties of tumorigenic cells by regulating NEGR1 expression. Proliferative and migratory abilities of adenocarcinoma cells are modulated by targeting the miR-576-5p–NEGR1 axis, which was suggested as a potential therapeutic objective for this disease [79]. However, the role of NEGR1-associated miRNAs as pro-apoptotic regulators is far from being clearly delineated. Indeed, contrasting data reported an inverse trend for miRNAs and NEGR1 in cell survival. In a study investigating the pathogenesis of vascular diseases, miR-25-5p exerted a neuroprotective role in human brain microvessel endothelial cells exposed to oxidised low-density lipoproteins, a significant risk factor for vascular diseases [80]. Increased miR-25-5p reduced pro-apoptotic proteins and ROS production while augmenting NO levels. Conversely, NEGR1 was overexpressed in treated cells, and its inhibition by RNA interference produced effects similar to miR-25-5p overexpression. Moreover, miR-25-5p overexpression and NEGR1 inhibition reduced p-STAT3 and p-JAK2 proteins, proving the involvement of this pathway in the miRna-25-5p–NEGR1 axis [80].

Another study identified a further pathway regulating NEGR1 expression, involving the circRNAs circHECW2 and miR-30e-5p, in the regulation of the endothelial–mesenchymal transition occurring in numerous dysfunctions of the blood–brain barrier [81]. CircHECW2 was significantly elevated in human brain microvascular endothelial cells treated with lipopolysaccharides, which stimulated proliferation and endothelial–mesenchymal transition. Using miRNA inhibitors and mimics, an interaction was demonstrated between circHECW2 and miR-30e-5p, whose levels were increased by circHECW2 inhibition. Moreover, circHECW2 silencing promoted cell proliferation and mitigated endothelial–mesenchymal transition and apoptosis. Dual-luciferase assays demonstrated NEGR1 to be a miR-30e-5p target protein, with very high levels after LPS treatment that were counteracted by circHECW2 silencing. Thus, mir-30e-5p was reported to inhibit endothelial–mesenchymal transition through the regulation of NEGR1 expression [81].

In a rat model of spinal cord ischaemia–reperfusion injury, the mutual interaction between the lncRNA MIAT (myocardial infarction-associated transcript), miR-150-5p, and their interaction with NEGR1 had similar effects on cellular apoptosis [82]. Luciferase assays showed that MIAT upregulated NEGR1 levels by competing with miR150-5p for binding to its promoter. MIAT inhibition increased miR-150-5p levels and reduced NEGR1, concurrently reducing pro-apoptotic proteins, increasing Bcl2 levels, and promoting a recovery of motor activity in behavioural tests [82].

Further miRNAs recognizing NEGR1 as their target have been predicted in different computational studies. Interactions between these molecular actors have been studied through analyses of the differentially expressed genes in several disease models. Associations between mRNAs and miRNAs were analysed in different types of glioblastoma [83]. The transcriptome from human glioblastoma samples was analysed to identify candidate miRNAs and mRNAs differently expressed from controls, studying the positive and negative correlations between them. It was observed that miR-21-5p was negatively correlated with NEGR1. Overexpression of this miRNA correlates with a later diagnosis, while that of NEGR1 correlates with an early diagnosis. Low levels of NEGR1 were associated with tumour recurrence, while low expression of miR-21-5p significantly stimulated apoptosis and reduced cell migration in vitro [83]. Similar experiments were performed to identify potential biomarkers for carotid atheromatous plaque, a risk factor for ischaemic stroke. Differentially expressed genes were identified and successively validated in the serum of acute ischaemic stroke patients. Among differentially expressed RNAs, associations were predicted between NEGR1 and miR-9, miR-181, and miR-124. Among them, miR-9 was proposed as a biomarker for acute ischaemic stroke [84]. In another study, differentially expressed RNAs were investigated in the maturation of mesenchymal stem cells, in order to define genes involved in the determination of osteoblast or adipocyte destiny. In this model, NEGR1 levels were upregulated during osteoblast differentiation, while its associated miRNA mir-203 and mir-382 were downregulated [85]. Lastly, the serum of patients at different stages of ovarian cancer was analysed to determine differently expressed miRNAs, which were correlated with disease biomarkers, including NEGR1. It was proposed that mir-4314 decreased the expression of GRWD1, IP6K1, and NEGR1, with a possible correlation with ovarian cancer onset [86].

Finally, a very recent study discovered that higher expression of the ferroptosis-related HMGA2-AS1 lncRNA was associated with worse clinical prognosis in bladder cancer patients. Remarkably, the ceRNA network analysis identified miR-181a-5p as a key signature component, modulating the expression of seven mRNAs, including NEGR1 [87].

## 7. IgLON5

In a study by Xiong et al. [88], the protective effects of the natural compound limonin was investigated on cardiac repair post-myocardial infarction, identifying IgLON5 as crucial in the cardioprotective effects of limonin. A central finding was the identification of the miR-10a-5p-IGLON5–LMX1A axis as a significant pathway in limonin-induced cardioprotection based on recent evidence showing that non-coding RNAs are critical in the pathophysiological processes of myocardial infarction [89]. Verification using RT-qPCR showed that myocardial infarction caused decreased miR-10a-5p expression and increased IGLON5 expression. Moreover, limonin administration reversed these changes, increasing miR-10a-5p levels and decreasing IGLON5 expression, enhancing cardiac repair mechanisms, reducing cardiomyocyte apoptosis, and improving ATP production [88].

## 8. Conclusions

The role of miRNAs in the regulation of the expression of IgLON cell adhesion molecules has been investigated in precious few studies, which however underlined their involvement in the precise functioning of these proteins. Indeed, alterations in specific miRNA expression were reflected in concurrent changes in IgLON levels, determined both in vitro and in silico in various biological conditions. Certainly, many more studies are necessary to unveil the precise regulation of these molecules and the pathways of association between them, but the interaction between miRNAs and IgLONs could represent a promising new target to characterise, endowed with potential therapeutic impact in many pathophysiological conditions. In particular, the available evidence supports a potential application of miRNA signatures as efficacy biomarkers able to predict prognostic outcomes and therapy response, thus contributing to the implementation of personalised medicine.

## Figures and Tables

**Table 1:** IgLON family members.

IgLON Name	Alias	Expression	Role
LSAMP	IgLON3, LAMP	Brain, retina-enriched, cancer-enriched	Cell adhesion molecule
OPCML	IgLON1, OBCAM	Brain, parathyroid gland, retina-enriched, cancer-enriched	Cell adhesion molecule, neuronal signalling
NTM	IgLON2, neurotrimin, CEPU-1	Brain, retina-enriched, cancer-enhanced	Cell adhesion molecule
NEGR1	IgLON4, kilon, neurotractin	Brain-enriched	Cell adhesion molecule, synaptic function
IgLON5		Brain, testis-enriched, cancer-enriched	Cell adhesion molecule, brain development

**Table 2:** miRNAs associated with each IgLON in different models, with the reported effects as described in following sections.

miRNA	Target	Model	Target Variation
miR-206	LSAMP	Nerve lesion	Potential regulatory role
miR-375	LSAMP-AS1	Tumour samples	Inhibition of miR-375 by upregulated LSAMP-AS1
miR-1193-3p; miR-129-1-3p; miR-129-2-3p; miR-132-3p; miR-1930-5p; miR-212-3p; miR-297a-3p; miR-297b-3p; miR-297c-3p; miR-3068-3p; miR-3069-3p; miR-3072-3p; miR-3102-3p; miR-326-3p; miR-329-3p; miR-350-3p; miR-369-3p; miR-434-3p; miR-450b-3p; miR-466a-3p; miR-466b-3p; miR-466c-3p; miR-466e-3p; miR-466f-3p; miR-466h-5p; miR-466p-3p; miR-467d-3p; miR-488-3p; miR-700-5p; miR-874-5p	LSAMP	HuD KO mice	Predicted LSAMP downregulation
miR-541-5p	LSAMP	Hypoxia-induced pulmonary hypertension	Predicted LSAMP downregulation
miR-129-5p	LSAMP-AS1	Pancreatic cancer	Regulating hypoxic tumoural environment
miR-4447	LSAMP	Tumorigenesis	miRNA encoded within LSAMP
miR-301b-3p	LSAMP	Hepatocellular carcinoma	Suppression of LSAMP gene expression
miR-29b-2-5p and miR-342-3p	LSAMP	Leukaemia/lymphoma	Interaction in the pathogenetic mechanism of the disease
miRNA-200c-3p	LSAMP	Colorectal cancer	Inhibition of LSAMP expression
miR-224-5p	OPCML	Gastric cancer	Downregulates OPCML
hsa-mir-137	OPCML	Gastric cancer	Downregulates OPCML via POU6F2-AS2
hsa-mir-372, hsa-mir-373, hsa-mir-519d, hsa-mir-184, hsa-mir-205, hsa-mir-506, hsa-mir-375	OPCML-iT1	Cholangiocarcinoma (CCA)	Predicted by miRcode database
MIR34B, Let-7a-3	OPCML	Epithelial ovarian cancer	Potentially influencing OPCML expression levels as methylation-sensitive
miR-184, miR-372, miR-373, miR-519d, miR-205, miR-375	OPCML-iT1	Thyroid cancer	Predicted by competing endogenous RNA (ceRNA) network
miR-758	NTM	Glioblastoma	Downregulation of NTM mRNA expression
miR-182	NTM	Peripheral nerve injury	Downregulates mRNA and NTM protein levels
miR-377	NEGR1	Neurodevelopment of primary cells/OGD	Upregulation of NEGR1 in neurodevelopment primary cells. Predicted interaction in OGD
miR-203	NEGR1	Neurodevelopment of primary cells/OGD	Downregulation of NEGR1 in OGD. Predicted interaction in neurodevelopment of primary cells
miR-181a-5p	NEGR1	OGD	Downregulation of NEGR1
miR-181a-5p	NEGR1	Bladder cancer	NEGR1 regulation
miR-503-3p	NEGR1	OGD	Downregulation of NEGR1
miR-576-5p	NEGR1	Colon adenocarcinoma	Downregulation of NEGR1
miR-25-5p	NEGR1	HBMEC treated with OX-LDL	Downregulation of NEGR1
miR-30e-5p	NEGR1	HBMEC treated with LPS	Downregulation of NEGR1
miR-150-5p	NEGR1	Spinal cord ischaemia–reperfusion injury	Downregulation of NEGR1
miR-21-5p	NEGR1	Glioblastoma	Predicted negative correlation with NEGR1
miR-9, miR-181, miR-124	NEGR1	Acute ischaemic stroke	Predicted interaction with NEGR1
miR-203, miR-382	NEGR1	Mesenchymal stem cells	Predicted interaction with NEGR1
miR-4314	NEGR1	Ovarian cancer	Predicted downregulation of NEGR1
rno-miR-10a-5p	IGLON5	Myocardial infarction (MI)	MI caused decreased miR-10a-5p expression and increased IGLON5 expression
